# Quercetin in Shengxian Decoction exhibits anti-ferroptosis protective roles in a myocardial infarction model *via* targeting DPP4/ HMOX1, based on network pharmacology and molecular docking

**DOI:** 10.3389/fphar.2025.1583509

**Published:** 2025-04-29

**Authors:** Yuming Zhai, Jiamei Fu, Jianfei Yang, Yabin Zhou

**Affiliations:** ^1^ Graduate School, Heilongjiang University of Chinese Medicine, Harbin, Heilongjiang, China; ^2^ Department of Cardiovascular Medicine, The First Affiliated Hospital of Heilongjiang University of Chinese Medicine, Harbin, Heilongjiang, China

**Keywords:** myocardial infarction, Shengxian Decoction, quercetin, ferroptosis, network pharmacology

## Abstract

**Background:**

Myocardial infarction (MI) is characterized by high morbidity. In this study, we aimed to elucidate potential targets of Shengxian Decoction (SXD) against MI.

**Methods:**

Pairing of SXD active ingredients and MI targets was conducted using the Chinese Medicine System Pharmacological Database, Gene Expression Omnibus (GEO), and STRING databases. The effects of SXD on MI were validated *in vitro*. Molecular docking was verified using cellular thermal shift assay (CETSA).

**Results:**

A total of 40 active ingredients and 28 MI-related targets were obtained. Cross-analysis on 28 targets and cell death-related genes identified two crucial ferroptosis-related targets, namely, dipeptidyl peptidase 4 (DPP4) and heme oxygenase 1 (HMOX1). In cobalt chloride (CoCl_2_)-induced hypoxic H9c2 cells, SXD could remarkably improve cell viability and inhibit cell death. Meanwhile, SXD treatment significantly affected the ferroptosis-related markers in hypoxic H9c2 cells. Molecular docking and CETSA results showed that quercetin had good binding activity with DPP4 and HMOX1.

**Conclusion:**

Important active ingredient quercetin in SXD could exert anti-ferroptosis protective roles on MI through targeting ferroptosis-related genes (DPP4/HMOX1), thereby contributing to the protective role of SXD on MI.

## 1 Introduction

Myocardial infarction (MI) is clinically defined as myocardial cell death due to prolonged ischemia, and patients are characterized by high morbidity, mortality, and disability rates (Lu et al., 2015; Hu and Peng, 2024). Typically, MI often originates from the coronary atherosclerotic thrombosis or myocardial oxygen imbalance (Yang et al., 2024). Acute MI (AMI) is caused by a long-lasting and severe myocardial ischemia of acute necrotic myocardium, and it is the most serious and dangerous form of MI, with high morbidity, dangerous conditions, and poor prognosis (Occhipinti et al., 2021). In recent years, the occurrence of AMI has increased significantly, along with the trend of rejuvenation, and it is expected that the number of AMI patients in China will reach 23,000,000 by 2030 (Jortveit et al., 2020). It has been noted that there is a time dependence in AMI salvage, with a 10% increase in patient mortality for every 1-h delay in reperfusion therapy (Esposito et al., 2018). MI leads to impaired cardiac systolic function, myocardial hypertrophy, fibrosis, and altered autophagic activity, ultimately resulting in ventricular dysfunction. Post-AMI, cardiomyocytes die in large numbers due to persistent ischemia and hypoxia, causing alterations in ventricular structure and function, and ultimately developing heart failure (Bahit et al., 2018; Chen et al., 2022). Currently, the main treatment for MI is reperfusion, and mitigating reperfusion injury is gradually becoming one of the key issues in the fight against MI. It is often treated clinically with antithrombotic drugs, coronary intervention, or bypass surgery (Granger et al., 1992; Pan et al., 2021). Traditional Chinese medicine (TCM) could reduce myocardial damage after MI in multiple ways, regulate emotions, reduce pain, and improve the quality of life, as well as have a preventive effect on MI and reduce the incidence of AMI (Spatz et al., 2018; Curfman, 2023).

Studies have shown the therapeutic effects of TCM on MI. For example, Shensong Yangxin Capsule has been shown to improve the myocardial blood flow and capillary endothelial structure, thereby alleviating MI (Jiang et al., 2021). Tongxinluo can significantly increase the left ventricular ejection fraction of MI patients, reduce the rate of rehospitalization of heart failure, and improve the clinical symptoms and cardiac function of patients (Yang Y. et al., 2023). Shengxian Decoction (SXD), a well-known TCM formula composed of *Bupleuri Radix* (Chaihu in Chinese, CH), *Cimicifugae Rhizoma* (Shengma in Chinese, SM), *Astragali Radix* (Huangqi in Chinese, HQ), *Platycodonis Radix* (Jiegeng in Chinese, JG), and *Anemarrhenae Rhizoma* (Zhimu in Chinese, ZM), is clinically considered an effective formula against cardiovascular diseases (Huang et al., 2021). Among them, Astragaloside IV (AS-IV), one of the main components of HQ, was able to improve AMI cardiac remodeling and reduce cardiomyocyte focal death. This might be related to the inhibition of ROS production and reduction in caspase-1 activity and GSDMD expression by AS-IV. Thus, it attenuated cardiomyocyte apoptosis and necrosis, inhibiting the occurrence of myocardial fibrosis and cardiac remodeling (Shi et al., 2021; Zhang et al., 2022; Yang L. et al., 2023). Saikosaponin D inhibited Nox4/PKCα/Gal-3 pathway activation, attenuating inflammatory injury of myocardial tissue, alleviating myocardial fibrosis, and improving cardiac function in MI rats (Liu et al., 2018).

Network pharmacology is an emerging interdisciplinary discipline combining systems biology and network informatics, which has been used widely in drug discovery and development in recent years (Zhao et al., 2023). Elucidating the mechanism of action by constructing relationships among drug chemistry, targets of action, and disease targets, systems biology suggested that complex diseases like MI are not affected by a single target gene. They are caused by mutations in multiple genes, leading to a disruption of the biological network system. The role of multiple drug components of SXD was analyzed at a systemic level through network pharmacology, which helped us find the active ingredients of MI therapeutic targets. Molecular docking is a computer simulation technique that simulates the interactions between molecules and protein at the atomic level, predicts the conformations of ligands and receptors, and calculates some parameters, such as affinity (Stanzione et al., 2021).

In this study, we utilized a network pharmacology approach to screen the active ingredients and potential targets of SXD in treating MI. We explored the potential mechanism of resisting ferroptosis and alleviating inflammation using the molecular docking technology, which would provide new ideas for the clinical management of MI.

## 2 Material and methods

### 2.1 Collection and screening of active compositions in SXD

The Chinese Medicine System Pharmacological Database and Analysis Platform (TCMSP, https://old.tcmsp-e.com/tcmsp.php) provides interactive data related to the relationships among drugs, targets, and diseases (Ru et al., 2014). The platform also provides information on the identification of active ingredients, herbal component–target interactions, and their pharmacokinetic effects such as drug-likeness (DL), oral bioavailability (OB), intestinal epithelial permeability, water solubility, and permeability (Sun et al., 2022). In this study, the TCMSP database was used to screen the potentially active compounds of SXD by setting OB ≥ 30% and DL ≥ 0.18 using the following keywords: CH, JG, SM, HQ, and ZM. OB described the ability of delivery of an orally administered drug to the somatic circulation. DL could help screen out drug candidates with better pharmacokinetic properties and safety so as to improve the success rate and efficiency of drug development (Ru et al., 2014). Compounds with reviewed OB and DL thresholds were selected as active compounds for further analysis. The target information was then obtained from the “Related Targets.”

### 2.2 MI-related differentially expressed gene analysis

The MI-related dataset (GSE66360) was downloaded from the Gene Expression Omnibus (GEO, http://www.ncbi.nlm.nih.gov/geo/) database. There were 49 MI samples and 50 healthy controls in the cohort (processed on GPL570 [HG-U133_Plus_2] Affymetrix Human Genome U133 Plus 2.0 Array).

Differentially expressed genes (DEGs) were screened between MI and control samples, based on the limma (Ritchie et al., 2015) function package of R software [version 3.52.4] with Log_2_ fold-change (FC) absolute value >0.6 and false discovery rate (FDR) < 0.05 as criteria (the Benjomini–Hochberg method was used for the correction).

### 2.3 “Component–disease target” network building

The “Component–disease target” network was built based on the shared targets between MI-related genes and predicted SXD targets, which were identified using the jvenn online tool (https://jvenn.toulouse.inrae.fr/app/example.html).

### 2.4 Protein–protein interaction network analysis of common targets between SXD and MI

The STRING is a database for analyzing and predicting the functional connection of proteins and protein interactions, and we used the STRING 11.0 (Szklarczyk et al., 2019) platform to analyze the functional linkages of protein–protein interaction (PPI) using Cytoscape3.7.2 (Shannon et al., 2003) to construct visual PPI networks. The core targets were filtered according to the size and color shade changes in the nodes, which also represented the degree value.

### 2.5 GO and KEGG enrichment analysis

We used the “clusterProfiler” (Yu et al., 2012) package in R software to perform Gene Ontology (GO) terms and Kyoto Encyclopedia of Genes and Genomes (KEGG) enrichment analysis. The significantly enriched GO term and KEGG pathway were screened by the criterion of p.adjust< 0.05 to predict the main potential targets of SXD and the role of the potential biological activity mechanism. P-value was adjusted using the Benjomini–Hochberg method.

### 2.6 Screening for SXD-regulated cell death modalities

Increasing evidence has suggested that cardiomyocyte apoptosis (Wang et al., 2016), pyroptosis (Zhong et al., 2023), ferroptosis (Shen et al., 2022), and necroptosis (Zhe-Wei et al., 2018) are important pathologic mechanisms in MI. We downloaded cell death marker genes from the Molecular Signatures Database (MSigDB) (http://www.broadinstitute.org/msigdb). Crucial cell death-related markers were determined after a cross-analysis.

### 2.7 Preparation of drug and reagents

#### 2.7.1 SXD extract

The SXD was prepared according to the previous report (Yang Z. Q. et al., 2023), weighing 22.4 g HQ, 11.2 g ZM, 5.6 g CH, 5.6 g JG, and 3.7 g SM. The herbs were soaked in ultrapure water (mass:volume = 1:10) for 1 h, which was then decocted for 40 min. After filtering and collecting the extract, the herbs were extracted again. Then, the filtrates were combined together and concentrated to a volume of approximately 200 mL. The 200-mL extract was transferred to an oven to dry at 40°C. Before subsequent experiment, the dry extract was diluted with ultrapure water to 2.04 g/mL.

#### 2.7.2 Cell culture medium

High-glucose Dulbecco’s modified Eagle medium (DMEM) (PM150210, Procell, Hubei, China) containing 10% fetal bovine serum (FBS) (164210, Procell) and 1% penicillin–streptomycin mixture (PB180120, Procell) was taken as the complete medium for subsequent cell culture, stored at 4°C.

#### 2.7.3 Cobalt chloride solution

A total of 95.2 mg of cobalt chloride (CoCl_2_) powder (F2116358, Aladdin, Shanghai, China) was fully dissolved in 1 mL sugar-free DMEM (PM150270, Procell), and the concentration of the stock CoCl_2_ solution was 400 mM (stored at −20°C). The stock CoCl_2_ solution could be diluted to 400 μM, 300 μM, 200 μM, and 100 μM when we used.

### 2.8 SXD drug-containing serum preparation

Two-month-old male SD rats were purchased from Beijing Sipeifu Bio-Technology Co., Ltd. [License No.: SCXK (Beijing) 2019–0010], with an average body weight of 200 ± 20 g. All rats were randomly divided into three groups, namely, control group, SXD low-dose group (SXD-L), and SXD high-dose group (SXD-H). Based on the previous literature, rats in SXD-L and SXD-H groups were gavaged with 10.2 g/kg/day and 20.4 g/kg/day SXD extracts for 7 days, respectively (these doses have been indicated to improve the cardiac function in MI rats) (Yang Z. Q. et al., 2023). Rats in the control group were gavaged with the same volume of saline. One hour after the final gavage, blood was collected from the abdominal aorta of rats (Liu et al., 2022a), which was then allowed to stand at room temperature for 30 min before centrifugation at 3,000 rmp for 10 min. After that, serum samples were obtained (three rats per group).

Freshly prepared rat serum samples were then inactivated in a water bath at 56°C for 30 min, which were then filtered with a 0.22-μm membrane filter. Finally, DMEM containing 10% rat serum and 1% penicillin–streptomycin mixture was taken as SXD drug-containing serum, stored at 4°C.

### 2.9 Cell culture and grouping

The H9c2 rat cardiomyocytes were purchased from the Wuhan Procell (CL-0089, Hubei, China). The well-grown H9c2 cells were then seeded in six-well culture dishes, which were divided into four groups, namely, control group (control), CoCl_2_-induced hypoxic H9c2 cell model group (model group), SXD-L group, and SXD-H group.

After overnight culture, all groups of cells were treated with CoCl_2_ to induce hypoxia (for 24 h), except for the control group. Then, different doses of SXD were used to treat cells for 12 h, after which cells were collected for subsequent experiments.

### 2.10 Construction of CoCl_2_-induced hypoxic H9c2 cells

In this study, hypoxia induced by CoCl_2_ was adopted, and CoCl_2_ was considered a common hypoxia-mimetic agent (Grochans et al., 2022). The following treatment was performed to determine the best treatment concentration of CoCl_2_ (Table 1) (Jeong et al., 2020).

**TABLE 1:** Detailed CoCl_2_ treatments of various groups.

Groups	Treatment
Control group	H9c2 cells were cultured with complete medium overnight
Model group	H9c2 cells were cultured with complete medium overnight, and then, the medium was replaced by serum-free and glucose-free medium containing 400 μM, 300 μM, 200 μM, and 100 μM CoCl_2_ for 24 h
Blank	Only CCK8 solutions with no cell

### 2.11 Cell viability analysis

#### 2.11.1 CCK8 assay

H9c2 cells from different groups were collected and seeded in 96-well plates with 4,000 cells per well, which were then incubated overnight. After removing the complete medium, various doses of SXD drug-containing serum was added to treat cells as described above. Next, we added the CCK-8 solution in each well (C0038, Beyotime, Shanghai, China). After incubation for 1 h, optical density OD450 was determined. The cell viability was calculated using the following formula: cell viability (%) = [A (treatment groups) − A (blank average)]/[A (control group) − A (blank average)] × 100.

#### 2.11.2 Calcein–AM/PI fluorescence staining assay

In brief, the assay was conducted using the Live/Dead Cell Double Staining Kit (KTA1001, Abbkine, Hubei, China), strictly following the manufacturer’s instructions. First, 10× assay buffer was diluted with deionized water to 1× assay buffer. A measure of 1 μL of LiveDye and 1 µL of NucleiDye were added per 1 mL assay buffer, which was taken as the staining solution. Then, cells were washed using PBS (P1020, Solarbio, Beijing, China) twice after SXD treatment, adding 0.5 mL of staining solution per well and incubating at 37°C in the dark for 20 min. After that, cells were washed using PBS twice again. Subsequently, cells were observed and photographed using a fluorescence microscope. Live cells could be stained with green fluorescent LiveDye (Ex/Em = 488/530 nm), and dead cells could be stained with red fluorescent NucleiDye (Ex/Em = 535/617 nm). The number of live/dead cells was calculated using ImageJ.

### 2.12 Real-time PCR (RT-PCR) analysis

Total RNA was extracted using the TRIzol method from various groups of cells (15596-026, Thermo Fisher Scientific, United States). Then, the total RNA was reverse transcribed to cDNA using MightyScript Plus First Strand cDNA Synthesis Master Mix (gDNA digester) (B639252, Sangon Biotech, Shanghai, China). SGExcel Universal SYBR qPCR Mix (B532958, Sangon Biotech) was used to perform qPCR on the ABI 7000 Real-Time PCR Detection system. The thermocycling conditions were as follows: pre-denaturation at 95°C for 30 s, 40 cycles of denaturation at 95°C for 10 s, and annealing at 60°C for 15 s. The primers are summarized in Table 2. Finally, relative expression levels were calculated according to the 2^−ΔΔCT^ method.

**TABLE 2:** Primers used in RT-PCR analysis.

Gene	Species	Sequence (5'->3')	
*Acsl4*	Rat	Forward	CAT​ATC​GCT​CTG​TCA​CGC​ACT​TC
		Reverse	GGC​TGT​CCT​TCT​TCC​CAA​ACT​TG
*Gpx4*	Rat	Forward	ATG​CCC​GAT​ACG​CCG​AGT​G
		Reverse	ATT​TCT​TGA​TTA​CTT​CCT​GGC​TCC​TG
*Dpp4*	Rat	Forward	AGA​CAC​TCC​TAC​ACG​GCT​TCA​TAC
		Reverse	TTG​TGA​CCT​TCT​TGT​GAC​CAT​GTG
*Hmox1*	Rat	Forward	GGA​AGA​GGA​GAT​AGA​GCG​AAA​CAA​G
		Reverse	TGG​CTG​GTG​TGT​AAG​GGA​TGG
*β-actin*	Rat	Forward	CTA​TCG​GCA​ATG​AGC​GGT​TCC
		Reverse	GCA​CTG​TGT​TGG​CAT​AGA​GGT​C

### 2.13 Western blot

The cell samples were digested and collected, and the RIPA lysate (R0020, Solarbio) was added to extract protein, which was quantified using the bicinchoninic acid (BCA) kit (P0011, Beyotime) (Wang et al., 2018). The proteins were separated using SDS-PAGE gels and then transferred onto a polyvinylidene fluoride (PVDF) membrane. Subsequently, the membrane was incubated with primary antibodies and secondary antibodies, strictly following the manufacturer’s protocols. The antibodies used here included rabbit anti-ACSL4 (A20414, ABclonal, Wuhan, China), rabbit anti-GPX4 (A11243, ABclonal), rabbit anti-heme oxygenase 1 (HMOX1) (BM4010, BOSTER, Hubei, China), rabbit anti-DPP4 (BM5083, BOSTER), mouse anti-β-tubulin (BM3877, BOSTER), goat anti-rabbit IgG H&L (HRP) (511203, Zenbio, Chengdu, China), and goat anti-mouse IgG H&L (HRP) (511103, Zenbio). The blots were visualized using the ChemiDoc XRS + system (Bio-RAD, United States).

### 2.14 Molecular docking

Molecular docking was performed using CB-Dock2 software (labshare.cn) (Liu et al., 2022b). The 3D structures of hub proteins were obtained by entering the protein IDs in the RCSB Protein Data Bank (PDB, http://www.rcsb.org/pdb/). The pharmacodynamic composition of SXD was analyzed to screen out its major constituent quercetin, which was molecularly docked with ferroptosis-related hub genes.

### 2.15 Cellular thermal shift assay (CETSA)

The cells were seeded with 3 × 10^6^ cells/well, and then incubated with DMEM and quercetin (10 μM, NO.849061-97-8, Macklin, Shanghai, China) for 3 h. Subsequently, the cell lysates were collected and were equally divided into five aliquots, which were then heated for 3 min at 44°C, 48°C, 52°C, 56°C, and 60°C, respectively. After cooling at room temperature, the lysates were centrifuged for 10 min at 12,000 rpm. Next, protein levels in supernatants were detected using Western blotting.

### 2.16 Statistical analysis

All experimental data were analyzed in SPSS 20.0. The results were displayed as the mean ± standard deviation (mean ± SD). The difference significance among multiple groups was determined using one way-ANOVA, and the subsequent LSD method or Tamhane’s T2 method was chosen according to the variance. The p < 0.05 was considered statistically significant.

## 3 Results

### 3.1 Identification of MI-related candidate targets

**FIGURE 1:**
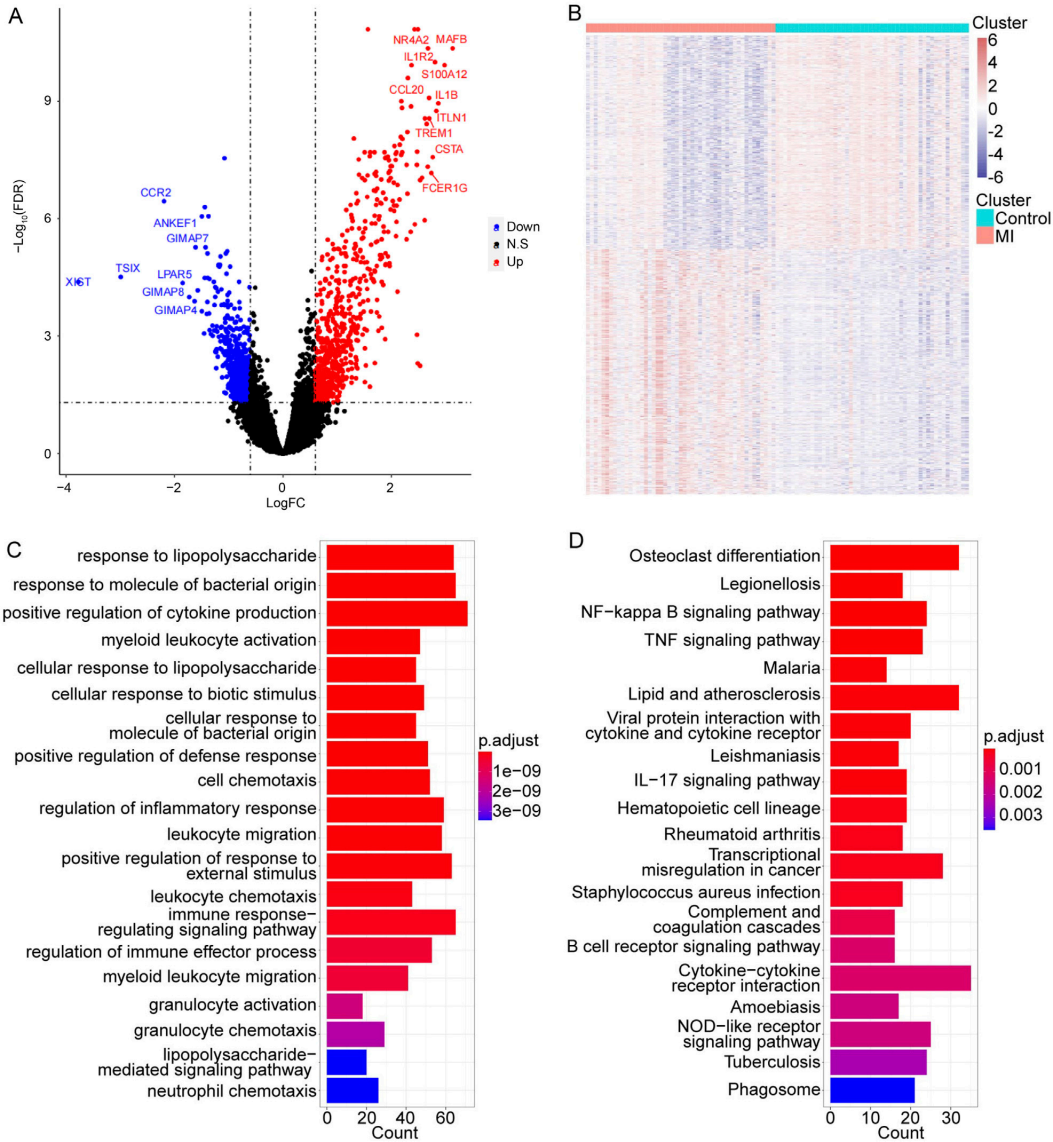
Identification of MI-related candidate targets and their functional information. **(A, B)** Volcano and heat map of 1,259 DEGs in MI. The top 10 significant gene symbols were displayed. **(C, D)** Top 20 significant GO and KEGG terms, separately.

To explore the crucial MI-related genes, the GSE66360 dataset was used to perform the DEG analysis. The results indicated that there were 672 upregulated genes and 587 downregulated genes in the MI samples compared to those in control samples (Figures 1A, B). The detailed gene list is displayed in Supplementary Table S1.

Subsequently, to further obtain more functional information on these MI-related genes, the GO and KEGG enrichment analyses were conducted. We found that these 1,259 DEGs were significantly enriched in a total of 599 GO terms and 31 pathways (detailed results are listed in Supplementary Table S2). The top 20 significantly enriched GO terms and 31 pathways are shown in Figures 1C, D.

### 3.2 SXD active ingredients and hub drug target identification

**FIGURE 2:**
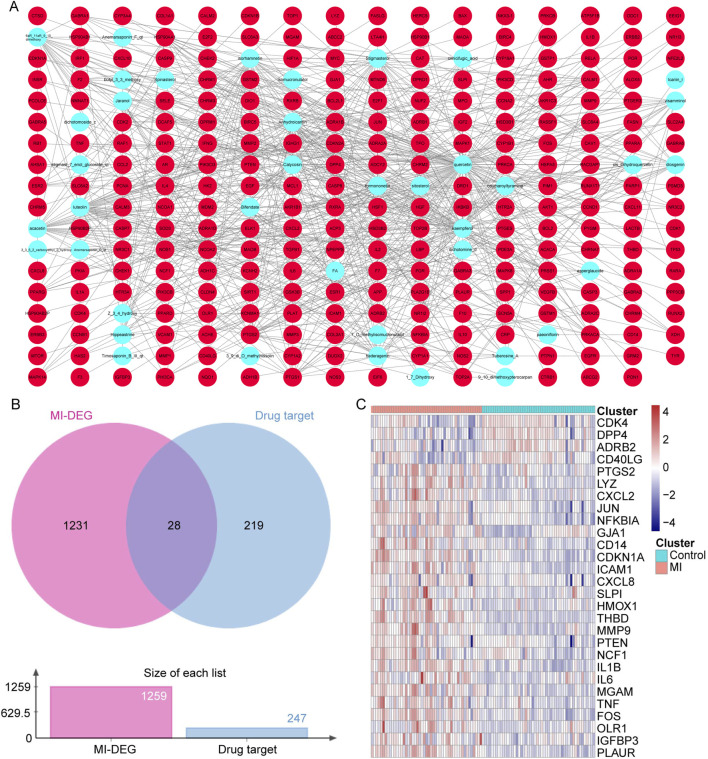
SXD active ingredients and hub drug target identification. **(A)** Compound–target network in SXD. **(B)** Venn diagram between unduplicated drug targets and MI-related genes. **(C)** Heat map of 28 candidate genes in the GSE66360 dataset.

To focus on the key active ingredients in SXD, the compounds of *Bupleuri Radix*, *Cimicifugae Rhizoma*, *Astragali Radix*, *Platycodonis Radix*, and *Anemarrhenae Rhizoma* were searched in the TCMSP database. Taking OB ≥ 30% and DL ≥ 18% as the criteria, a total of 40 unduplicated active ingredients were screened, which corresponded to 247 unique targets (Supplementary Table S3). After visualization of the interaction between active ingredients and drug targets, we found that quercetin from Astragalus targeted the most drug targets (over 150 targets) (Figure 2A).

Next, the 247 unduplicated drug targets were cross-analyzed with the 1,259 MI-related genes, and a total of 28 overlapped candidate genes were identified (Figure 2B). The expressions of these 28 candidate genes were also analyzed using the GSE66360 dataset (Figure 2C).

### 3.3 PPI network construction based on hub targets

**FIGURE 3:**
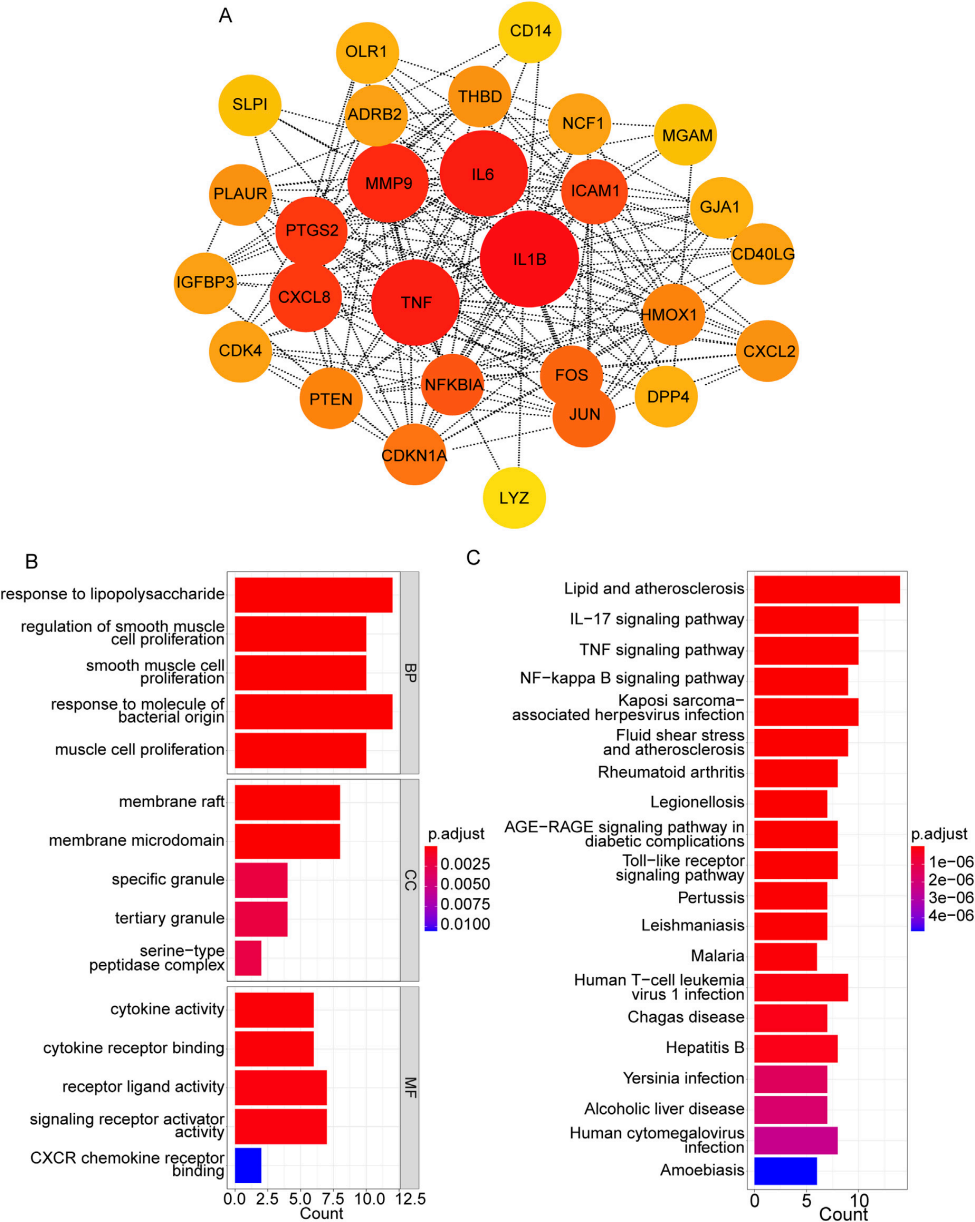
PPI network construction based on hub targets. **(A)** PPI network based on 28 target genes. **(B, C)** Top 15 significant GO terms **(B)** and top 20 significant pathways **(C)**.

Using the STRING database, the PPI network was constructed based on these 28 target genes and visualized using Cytoscape. The results showed that there were 28 nodes and 192 edges in the PPI network (each node represents a gene, and the edges represent the interactions between them) (Figure 3A). Using the MNC algorithm in the CytoHubba plugin, we found that the IL-1β gene has the highest connectivity (Table 3).

**TABLE 3:** Top 28 target genes in the PPI network.

Rank	Candidate target	Score	Rank	Candidate target	Score
1	IL1B	27	14	PLAUR	11
2	IL6	26	14	THBD	11
2	TNF	26	17	ADRB2	10
4	MMP9	25	17	IGFBP3	10
5	PTGS2	21	17	NCF1	10
5	CXCL8	21	17	CD40LG	10
7	ICAM1	20	17	CDK4	10
8	NFKBIA	18	22	GJA1	8
9	FOS	17	22	DPP4	8
9	JUN	17	22	OLR1	8
11	CDKN1A	15	25	MGAM	5
12	PTEN	14	25	SLPI	5
12	HMOX1	14	27	CD14	4
14	CXCL2	11	28	LYZ	2

**FIGURE 4:**
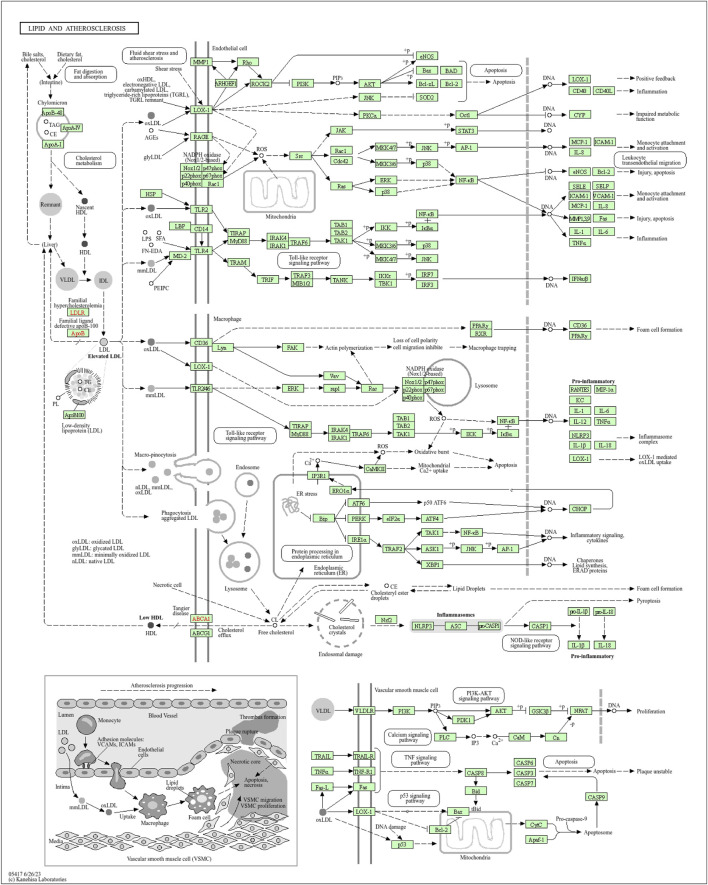
Pathway diagram of lipid and atherosclerosis (available at https://www.kegg.jp).

**FIGURE 5:**
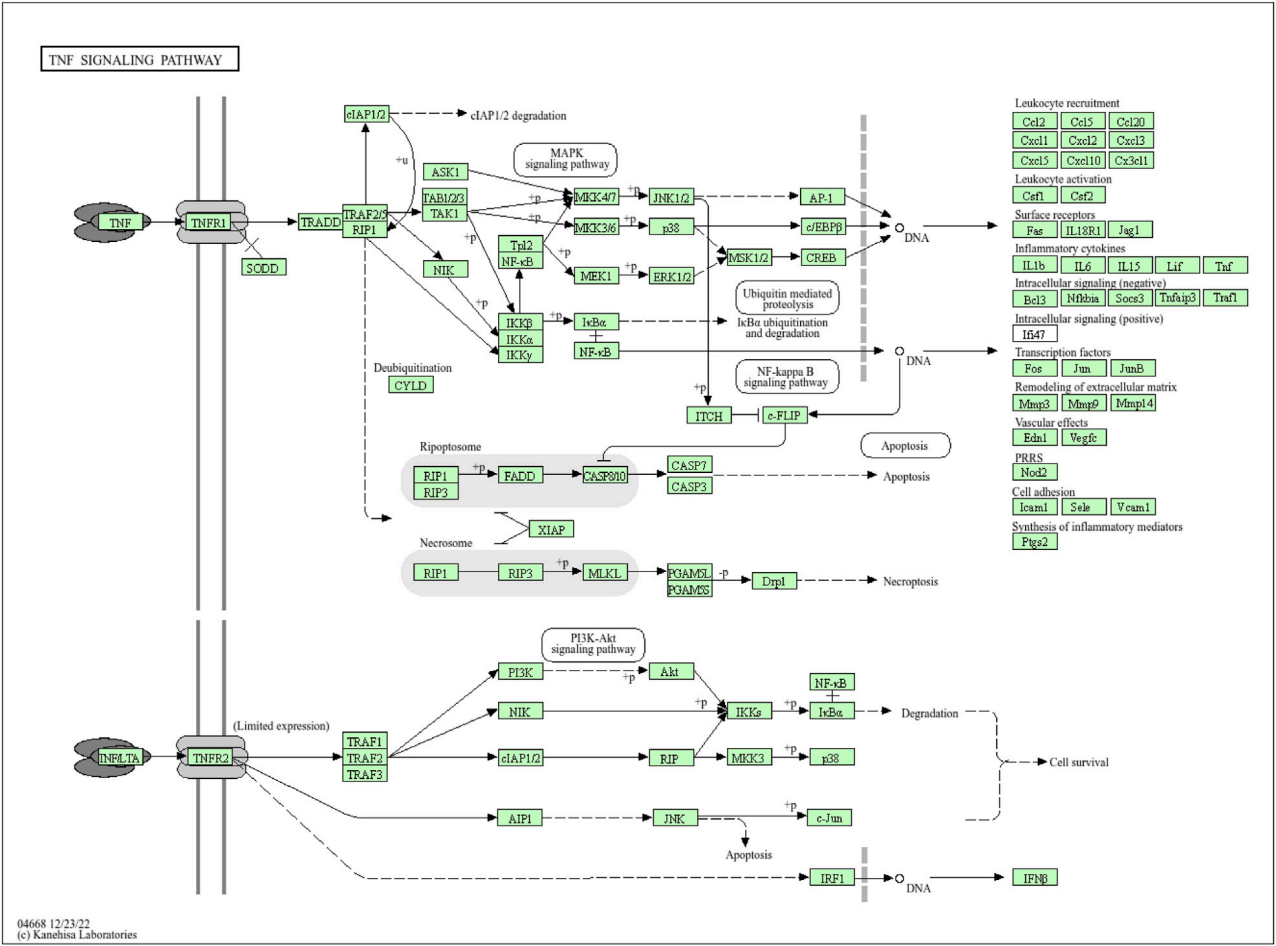
Pathway diagram of the NF signaling pathway (available at https://www.kegg.jp).

Moreover, the functional enrichment analysis suggested that 28 candidate genes were significantly enriched in 887 GO terms and 98 pathways (Supplementary Table S4), and the top 15 GO terms (Figure 3B) and top 20 pathways (Figure 3C) were exhibited. Of them, we noticed that several crucial pathways involving MI were significantly enriched, such as lipid and atherosclerosis (Figure 4) and the TNF signaling pathway (Figure 5).

### 3.4 Hub cell death-related genes in MI

**FIGURE 6:**
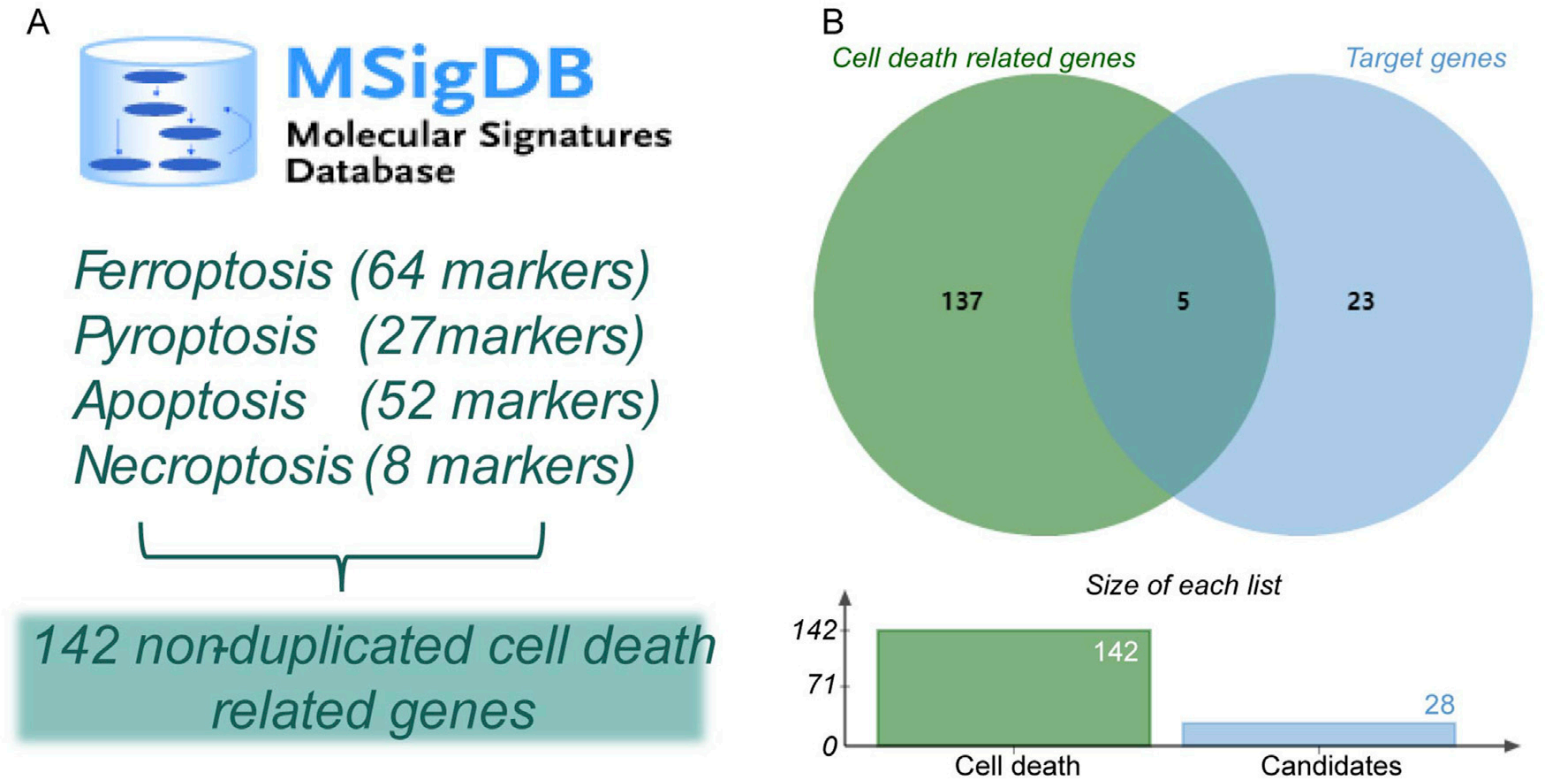
Identification of hub cell death-related genes in MI. **(A)** Identification of non-duplicated cell death-related genes. **(B)** Venn diagram of hub cell death-related genes in MI.

Considering the crucial impacts of various cell deaths on MI, we downloaded 64 ferroptosis-related genes, 27 pyroptosis-related genes, 52 apoptosis-related genes, and eight necroptosis-related genes from the MSigDB database (Table 4). We found a total of 142 non-duplicated cell death-related genes (Figure 6A). After the cross-analysis, five overlap genes were found between 142 cell death-related genes and 28 target genes, namely, dipeptidyl peptidase 4 (DPP4; ferroptosis), heme oxygenase 1 (HMOX1; ferroptosis), IL-1β (pyroptosis), TNF (necroptosis), and NFKBIA (apoptosis) (Figure 6B).

**TABLE 4:** Regulatory cell death-related genes.

Ferroptosis-related genes							
LPCAT3	DPP4	ALOX15	GPX4	SLC11A2	STEAP3	TF	SLC38A1
ATG7	ACSL1	FTH1	GSS	NOX4	CISD1	TFRC	MAP1LC3B
SAT2	ACSL3	FTL	SLC40A1	PCBP1	PRNP	TP53	MAP1LC3A
CP	ACSL4	GCH1	HMGCR	PCBP2	BACH1	TXNRD1	AIFM2
CTH	FDFT1	NOX1	HMOX1	CHMP5	SAT1	VDAC2	AKR1C3
CYBB	ACSL6	COQ2	HSPB1	ACSL5	SLC39A8	VDAC3	CBS
AKR1C1	SLC39A14	GCLC	IREB2	PHKG2	SLC1A5	CHMP6	FTMT
AKR1C2	SLC7A11	GCLM	MAP1LC3C	POR	SLC3A2	NCOA4	ATG5
Pyroptosis-related genes							
BAK1	GZMB	IL1A	CHMP2A	CHMP7	IRF2	CASP4	HMGB1
TP63	CHMP4B	CHMP3	CASP1	IL18	CYCS	ELANE	CHMP4C
CHMP2B	GSDMD	IRF1	CASP5	CASP3	CHMP6	CHMP4A	TP53
BAX	GSDME	IL1B					
Apoptosis-related genes							
PIK3R1	FOXO3	CASP8	LMNB1	CASP9	LMNB2	NFKB1	MAP3K14
PIK3CA	PIK3CG	PRF1	CASP10	LMNA	BCL2	TNFSF10	NFKBIA
FASLG	YWHAH	APAF1	CASP1	PARP1	TNFRSF25	BID	TRAF2
CHRNB1	PTK2B	BIRC3	CASP2	GZMB	CFLAR	CHUK	
AKT1	BAD	BIRC2	CASP4	CASP3	SPTAN1	TRADD	
MUSK	RAPSN	XIAP	CASP6	DFFA	GAS2	RIPK1	
TERT	CHRNG	ARHGDIB	CASP7	DFFB	RELA	FADD	
Necroptosis-related genes							
RIPK3	MLKL	FAS	FASLG	TLR3	TNF	RIPK1	FADD

Among the hub cell death-related genes in MI, two ferroptosis-related genes attracted our attention. DPP4, also known as CD26, is a type II transmembrane glycoprotein widely expressed in a variety of organs and cells. DPP4 binds with NOX1 to form the NOX1–DPP4 complex mediating the abnormal accumulation of intracellular iron ions and the onset of oxidative stress, making the cell more susceptible to injury by ferroptosis. HMOX1 degrades heme and releases free iron associated with oxidative stress and cardiac injury-related free iron and induces ferroptosis in cells.

### 3.5 SXD significantly inhibited the *in vitro* cell death of MI

**FIGURE 7:**
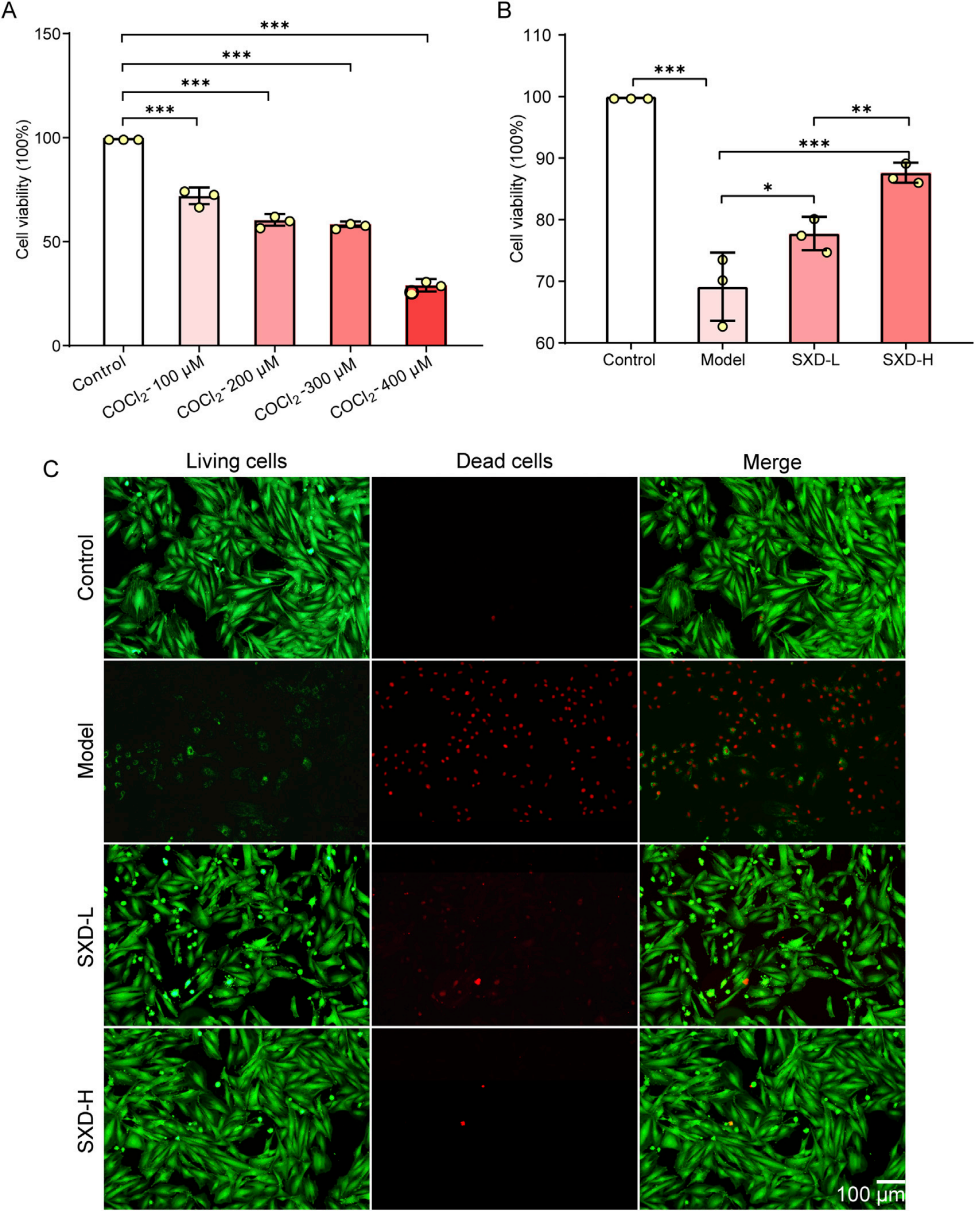
SXD significantly alleviated the cell death *in vitro*. **(A)** Evaluation of CoCl_2_ treatment concentration in H9c2 cells. **(B)** Effects of SXD on hypoxic H9c2 cells. *P < 0.05, **P < 0.01, and ***P < 0.001. **(C)** Results of calcein–AM/PI fluorescence staining in various H9c2 cells. Red fluorescence: dead cells; green fluorescence: alive cells.

To further validate the crucial effects of SXD on MI *in vitro*, we have then constructed the hypoxic myocardial cells. Typically, CoCl_2_ was established as a hypoxia-mimetic agent (Munoz-Sanchez and Chanez-Cardenas, 2019). After treating H9c2 cells with various concentrations of CoCl_2_, we found that the viability of H9c2 cells was significantly decreased when CoCl_2_ was over 200 μM, leading to severe cell death (Figure 7A). Hence, 100 μM CoCl_2_ inducing for 24 h was taken as the condition for generating CoCl_2_-induced hypoxic H9c2 cells.

Then, the effects of SXD on hypoxic H9c2 cells were determined using the CCK8 assay. The results showed that SXD could alleviate the poor cell viability induced by CoCl_2_, and the impacts of SXD were dose-dependent (Figure 7B). Moreover, the calcein–AM/PI fluorescence staining was used to explore the effects of SXD-L and SXD-H on cell death of hypoxic H9c2 cells. Compared to the model group, the dead cells significantly reduced after treating with a low/high dose of SXD (Figure 7C). Collectively, SXD could remarkably improve the cell viability and inhibit the *in vitro* cell death of hypoxic H9c2 cells.

### 3.6 SXD remarkably affected the ferroptosis-related markers in CoCl2-induced hypoxic H9c2 cells

**FIGURE 8:**
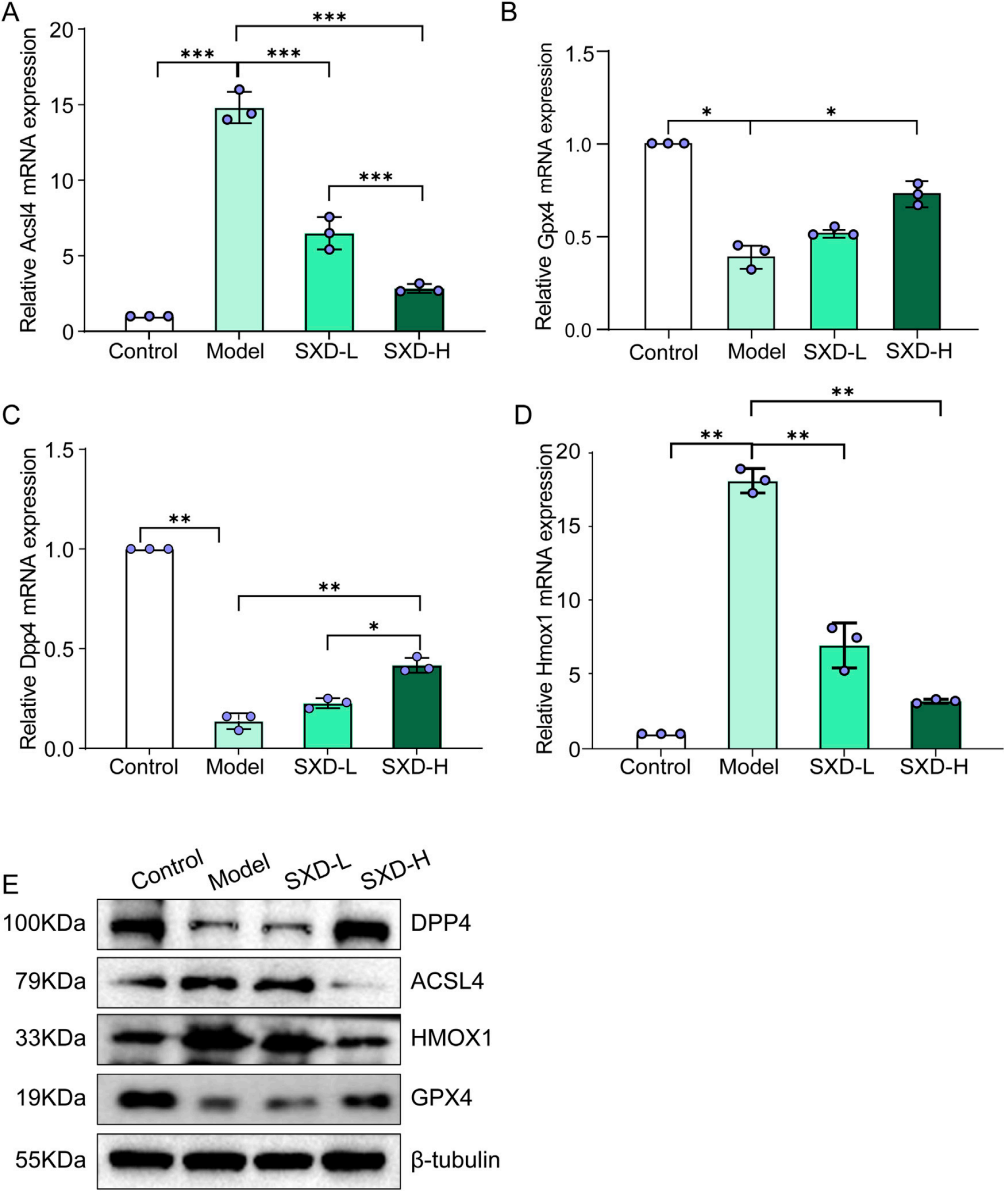
SXD remarkably affected the ferroptosis-related markers in CoCl_2_-induced hypoxic H9c2 cells. **(A–D)** mRNA expressions of ACSL4, GPX4, DPP4, and HMOX1 in different groups, separately. **(E)** Protein expressions of ACSL4, GPX4, DPP4, and HMOX1 in different groups. *P < 0.05, **P < 0.01, and ***P < 0.001.

Based on our *in silico* results and *in vitro* validation, important favorable effects of SXD on MI have been found. Meanwhile, two crucial ferroptosis-related genes were also identified, namely, DPP4 and HMOX1. Meanwhile, to further confirm the involvement of ferroptosis in the protective effects of SXD on MI, two established ferroptosis markers, ACSL4 and GPX4 (Moretti et al., 2024), were also included in our subsequent *in vitro* validation. Thus, the ferroptosis markers and these two hub genes were analyzed in various groups. Compared to the control group, ferroptosis markers, ACSL4 and GPX4, significantly upregulated/downregulated in hypoxic H9c2 cells, whereas SXD treatment significantly reversed their changes from both mRNA and protein levels (Figures 8A, B, E). Our data indicated that SXD was probably able to inhibit ferroptosis induced by hypoxia in H9c2 cells. Additionally, the mRNA and protein expressions of DPP4 and HMOX1 were also consistent with those in our findings based on the GEO dataset (Figures 8C–E).

### 3.7 Quercetin in SXD could target DPP4 and HMOX1 in MI

**FIGURE 9:**
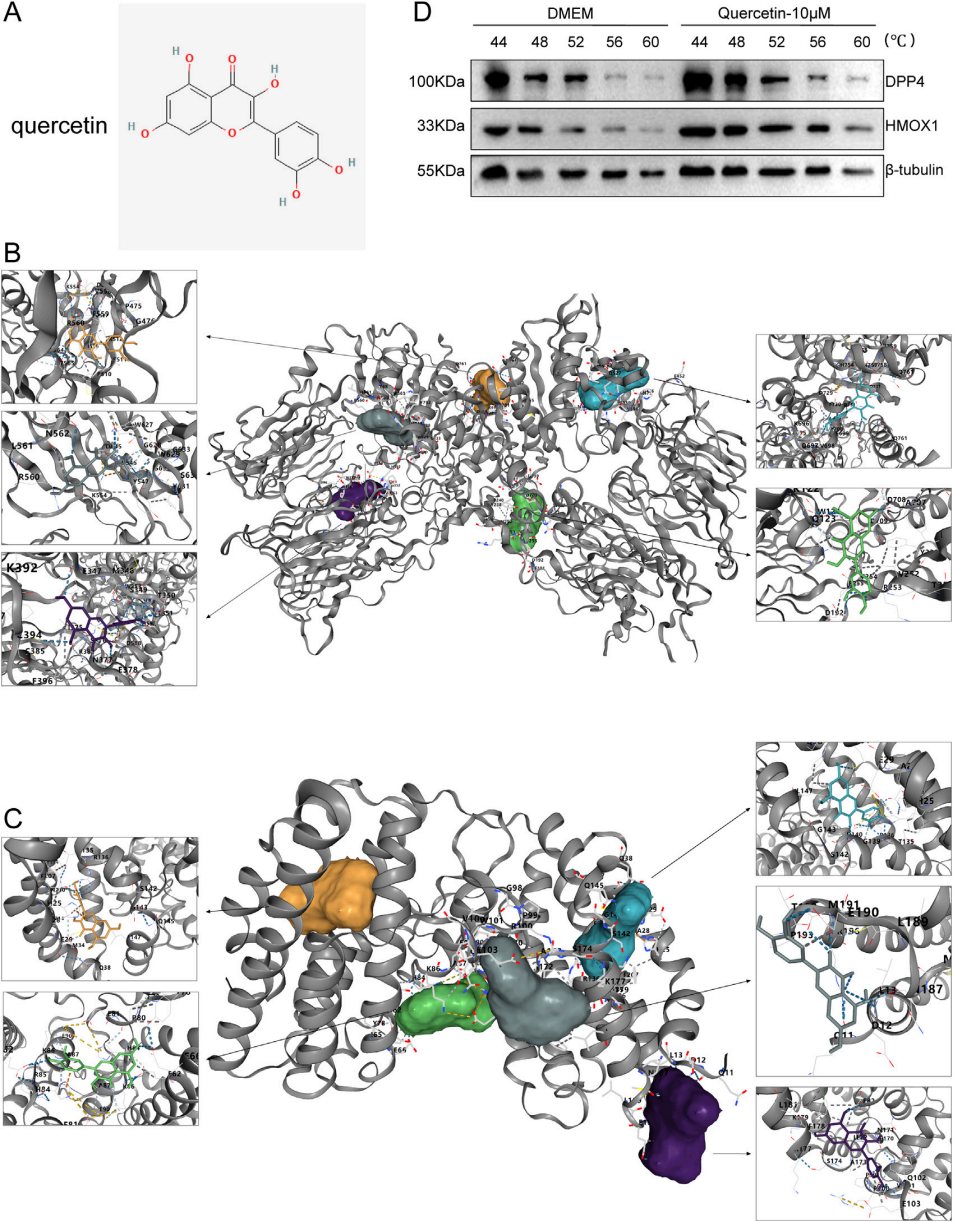
Molecular docking results. **(A)** Structure of quercetin. **(B, C)** Molecular docking of quercetin with DPP4 **(B)** and HMOX1 **(C)**. **(D)** Results of the CETSA assay.

Then, the active ingredient (quercetin) targeting most genes in SXD was selected for our subsequent molecular docking analysis (Figure 9A). The binding activity between quercetin and two ferroptosis-related genes was predicted, and the binding activity was evaluated using binding energy. Typically, the binding energy < −5 kcal/mol indicated a good binding activity. We found that there was binding activity between quercetin with DPP4 (Figure 9B) and HMOX1 (Figure 9C). The detailed docking information was displayed in Table 5.

**TABLE 5:** Detailed molecular docking results of DPP4 and HMOX1.

Target	Compound	CurPocket ID	Vina score	Docking size (x, y, z)
DPP4	Quercetin	C4	−8.1	21, 21, 21
		C2	−8	21, 21, 21
		C1	−7.6	27, 21, 21
		C3	−7.4	21, 27, 21
		C5	−7.1	21, 21, 21
HMOX1	Quercetin	C1	−7.5	21, 30, 21
		C2	−7.4	21, 30, 31
		C3	−6.8	21, 21, 21
		C5	−6.4	21, 21, 21
		C4	−5.5	21, 21, 21

Furthermore, we have used the CETSA assay to further validate the binding status of quercetin with DPP4 and HMOX1. In the DMEM control group, DPP4 (significantly degraded at 56°C) and HMOX1 (significantly degraded at 52°C) were gradually degraded along with the increasing incubation temperatures (Figure 9D). Meanwhile, after quercetin treatment (10 μM), both DPP4 and HMOX1 proteins remained almost undegraded at 56°C but were degraded at 60°C (Figure 9D). The results suggested that quercetin was able to bind to DDP4 and HMOX1, which would stabilize the target proteins at 56°C. Thus, active ingredient quercetin probably played a crucial role in MI through targeting DPP4 and HMOX1.

## 4 Discussion

The pathogenesis of MI is complex, including atherosclerotic plaque formation, inflammation, coronary artery stenosis, hypoxia and energy metabolism disorders, apoptosis, and fibrosis (Sousa Fialho et al., 2019; Marston et al., 2022; Cai et al., 2023; Fei et al., 2021). Studies have shown that ferroptosis is involved in multiple pathologic processes in MI, which might be a new target for the study of MI (Docherty et al., 2022; Miyamoto et al., 2022). The efficacy of TCM is the result of multi-targets, multi-pathways, and multiple active substances, which have unique advantages in regulating ferroptosis. Network pharmacology is characterized by systemic and holistic nature, which analyzes the complexity of TCM through the “multi-component, multi-target-multi pathway” interaction network, and provides a theoretical basis for clinical prevention and treatment (Jiashuo et al., 2022). In this study, the potential molecular mechanism of SXD in treating MI was revealed through network pharmacology, molecular docking, various databases, and *in vitro* experiments.

The active ingredient targets in SXD were screened in TCMSP, and a total of 247 targets were found. These targets were then subjected to an intersection operation with the 1,259 targets of MI, yielding a total of 28 targets for drug–disease intersection. After visualization and analysis using Cytoscape 3.7.2, the gene connectivity values were more than two times of the average node, and they were considered to be important targets for SXD for treating MI. Subsequently, the core targets were analyzed for changes in size and brightness by further analysis of PPI and connectivity analysis, and we found that SXD might be realized by interfering with a series of targets and pathways, such as IL-1β, IL-6, MMP9, PTGS2, CXCL8, ICAM1, HMOX1, and DPP4. After the cross-analysis of candidate targets and cell death-related markers, ferroptosis-related genes HMOX1 and DPP4 attracted our attention.

HMOX1 and DPP4 targets are important genes for ferroptosis and are involved in various physiological and pathological processes by regulating the energy metabolism and immune function (Fang et al., 2019; Ren et al., 2022). IL-1β and IL-6 are key genes in the inflammatory response. SXD active ingredients could act on these genes to exert their effects (Ridker and Rane, 2021). Meanwhile, ferroptosis is a regulated cellular necrosis caused by the excessive accumulation of iron-dependent lipid peroxides (Wang et al., 2023). Holay et al. (2012) found that serum ferritin levels and transferrin saturation were positively correlated with the incidence of MI and peripheral vascular disease. Park et al. (2019) have shown that ferroptosis occurs in cardiomyocytes during MI. Nrf2 is an important transcription factor that is primarily responsible for regulating cellular antioxidant responses. It helps cells resist ROS damage and apoptosis of iron cells in various cell types by activating the expression of a series of antioxidant enzymes and proteins. The activation of the Nrf2/HMOX1 pathway in the early and middle stages of MI causes iron overload, which leads to cardiomyocyte ferroptosis. Regarding DPP4, Krijnen et al. have documented that the expression and activity of DPP4 was significantly reduced in the infarction area of AMI samples (Krijnen et al. 2012), which was in line our findings.

It has been found that inhibition of the IL-1β intrinsic immune pathway is closely related to the anti-inflammatory effect and reduction of MI. After MI, hypoxia-induced injury to cardiac fibroblasts (CFs) causes increased secretion of NLRP3 in CFs and macrophages, inducing caspase-1 activation, which further leads to IL-1β secretion (Han et al., 2020). IL-1β promotes low-density lipoprotein deposition into the vessel wall. Macrophages phagocytose lipoproteins and turn into foam cells. The activation of the foam cell further promotes IL-1β and IL-6 secretion, leading to increased procoagulant activity and adhesion molecule expression, mononuclear phagocyte aggregation, and enhanced local inflammatory response. IL-1β acts on vascular smooth muscle cells to promote their proliferation, leading to intimal thickening, and can activate platelets, leading to necrosis of vascular smooth muscle cells (Fernandez et al., 2019; Abbate et al., 2020). The abovementioned mechanisms form a cyclic reaction that could lead to plaque enlargement and lower stability, which is more likely to lead to plaque rupture and promote thrombus formation, thus triggering AMI.

Studies have confirmed the efficacy of Chinese medicine compounds in the treatment of MI, such as Shensong Yangxin Capsule and Tongxinluo (Jiang et al., 2021; Yang Y. et al., 2023). SXD is based on Buzhongyiqi Decoction with addition, subtraction, and customization. HQ tonifies the vital energy and is the monarch drug. In our work, quercetin, the active ingredient of HQ, targeted most genes in MI. ZM is used to neutralize the warm and dry nature of HQ and is used as the assistant drug. SM, CH, and JG can elevate Yang. The combination of all the herbs played a role in benefiting qi and elevating trapping. The GO enrichment analysis of drug–disease intersection targets yielded 15 biological processes with the highest enrichment values. The most similar processes for SXD-treating MI included response to lipopolysaccharide, smooth muscle cell proliferation, muscle cell proliferation, membrane raft, response to molecule of bacterial origin, regulation of smooth muscle cell proliferation, and membrane microdomain. KEGG enrichment analysis revealed several disease-related pathways, including lipid and atherosclerosis, IL−17 pathway, TNF pathway, NF−κB pathway, Kaposi sarcoma-associated herpesvirus infection, fluid shear stress, and atherosclerosis. Moreover, the molecular docking results showed that the binding energies of quercetin to HMOX1 and DPP4 were all less than −5 kcal/mol, suggesting its potential role in the treatment of MI. Collectively, quercetin in SXD might exert crucial roles in regulating ferroptosis-related markers HMOX1 and DPP4, thereby against MI.

Although we have first revealed the potential protective targets of SXD in MI, there were still some limitations in this work. First, despite multiple publicly available databases, powerful bioinformatics tools, and *in vitro* validation, further *in vivo* studies would improve the robustness of our findings. For instance, the effects of SXD should be evaluated in MI animal models to confirm the *in vivo* efficacy of SXD. Moreover, our present work preliminarily explored the functional targets of quercetin, whereas deepening mechanisms underlying the protective effects on MI should be further deciphered. Meanwhile, the present data involving ferroptosis in the protective role of SXD in MI were limited, and more deepening investigation would be helpful for the understanding and application of SXD in MI. Based on this, further mechanistic details involving SXD and MI will be investigated in the near future. The validation in MI animal models and deepening mechanistic exploration will be prioritized in our follow-up work.

## 5 Conclusion

To summarize, our present work has focused on the protective role of SXD in MI, and the important active ingredient quercetin in SXD has exhibited great potential in targeting ferroptosis-related genes, DPP4 and HMOX1, thereby contributing to the favorable effects of SXD on MI. Our data have provided valuable insights for the application of SXD in MI, from both *in silico* and *in vitro* levels.

## Data Availability

The data presented in the study are deposited in the Gene Expression Omnibus repository (http://www.ncbi.nlm.nih.gov/geo/), accession number GSE66360.
